# B‐cell performance in chemotherapy: Unravelling the mystery of B‐cell therapeutic potential

**DOI:** 10.1002/ctm2.1761

**Published:** 2024-07-12

**Authors:** Zizhuo Li, Anqi Lin, Zhifei Gao, Aimin Jiang, Minying Xiong, Jiapeng Song, Zaoqu Liu, Quan Cheng, Jian Zhang, Peng Luo

**Affiliations:** ^1^ Department of Oncology Zhujiang Hospital Southern Medical University Guangzhou Guangdong China; ^2^ The First School of Clinical Medicine Southern Medical University Guangzhou Guangdong China; ^3^ Department of Urology Changhai Hospital Naval Medical University (Second Military Medical University) Shanghai China; ^4^ School of Basic Medical Sciences Xi'an Jiaotong University Xi'an Shaanxi China; ^5^ Institute of Basic Medical Sciences Chinese Academy of Medical Sciences and Peking Union Medical College Beijing China; ^6^ Department of Neurosurgery Xiangya Hospital Central South University Changsha Hunan China; ^7^ National Clinical Research Center for Geriatric Disorders Xiangya Hospital Central South University Changsha Hunan China

**Keywords:** anti‐tumour therapy, B cells, chemotherapy, neoadjuvant chemotherapy (NACT), targeting B cells, tumour microenvironment (TME)

## Abstract

**Background and main body:**

The anti‐tumour and tumour‐promoting roles of B cells in the tumour microenvironment (TME) have gained considerable attention in recent years. As essential orchestrators of humoral immunity, B cells potentially play a crucial role in anti‐tumour therapies. Chemotherapy, a mainstay in cancer treatment, influences the proliferation and function of diverse B‐cell subsets and their crosstalk with the TME. Modulating B‐cell function by targeting B cells or their associated cells may enhance chemotherapy efficacy, presenting a promising avenue for future targeted therapy investigations.

**Conclusion:**

This review explores the intricate interplay between chemotherapy and B cells, underscoring the pivotal role of B cells in chemotherapy treatment. We summarise promising B‐cell‐related therapeutic targets, illustrating the immense potential of B cells in anti‐tumour therapy. Our work lays a theoretical foundation for harnessing B cells in chemotherapy and combination strategies for cancer treatment.

**Key points:**

- Chemotherapy can inhibit B‐cell proliferation and alter subset distributions and functions, including factor secretion, receptor signalling, and costimulation.
- Chemotherapy can modulate complex B‐cell–T‐cell interactions with variable effects on anti‐tumour immunity.
- Targeting B‐cell surface markers or signalling improves chemotherapy responses, blocks immune evasion and inhibits tumour growth.
- Critical knowledge gaps remain regarding B‐cell interactions in TME, B‐cell chemoresistance mechanisms, TLS biology, heterogeneity, spatial distributions, chemotherapy drug selection and B‐cell targets that future studies should address.

## Graphical abstract

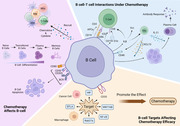

## 1 INTRODUCTION

In recent years, the tumour microenvironment (TME) has been identified as a crucial regulator of tumour progression and immune responses. The TME is composed of surrounding immune cells, blood vessels, fibroblasts, signalling molecules, bone marrow‐derived inflammatory cells and the extracellular matrix (ECM). Far from being a passive bystander, the TME actively promotes cancer progression, which is analogous to the relationship between seed and soil.^1^ Tumours exert influence on the TME by inducing angiogenesis and immune tolerance, and immune cells play a critical role in tumour growth.

The anti‐tumour and pro‐tumour functions of B cells in the TME have garnered significant attention, establishing B cells as emerging key players in cancer therapy. The presence of B cells in the TME is correlated with improved outcomes, which can be attributed to tumour‐specific antibody production, T‐cell activation and direct tumour cell lysis.^2^, ^3^, ^4^, ^5^, ^6^ Moreover, B‐cell‐associated pathways, such as CCL19/21‐CCR7 and CXCL13‐CXCR5, facilitate immune activation through humoral immunity and the formation of tertiary lymphoid structures (TLSs).^7^ However, pro‐tumourigenic B‐cell subsets, such as regulatory B cells (Bregs), can also promote immunosuppression and tumour progression through secreting cytokines, including IL‐10, TGF‐β and IL‐35.^2^, ^8^, ^9^, ^10^, ^11^ Therefore, the therapeutic potential of B cells merits further investigation.

Chemotherapy remains a cornerstone treatment for various cancers, including liver,^12^ lung,^13^ breast^14^ and colorectal cancer.^15^ Chemotherapy influences B‐cell numbers and function within the TME,^16^, ^17^, ^18^, ^19^, ^20^, ^21^, ^22^ thereby modulating anti‐tumour immunity and treatment efficacy. Most studies indicate that chemotherapy induces B‐cell reduction^17^, ^18^, ^19^; however, some reports show no change^23^ or increased B‐cell infiltration.^21^, ^24^ Furthermore, chemotherapy alters the ratios of B‐cell subsets,^16^, ^25^, ^26^ frequently elevating naïve B cells while decreasing memory B cells(MBCs).^26^ Moreover, chemotherapy may modify B‐cell function and the composition of the TME.

The associations between B cells and chemotherapy prognosis suggest that B cells could potentially serve as biomarkers. Higher levels of ICOSL+ B cells postchemotherapy are predictive of improved survival in breast cancer patients.^27^ The presence of plasma cells (PCs) indicates a better prognosis in hormone receptor‐negative breast cancer.^28^ Lower B‐cell levels are associated with poorer survival outcomes in ovarian cancer patients undergoing chemotherapy.^29^ A higher number of follicular B(FO‐B) cells is linked to long‐term survival in nonsmall cell lung cancer (NSCLC) patients receiving chemotherapy.^30^ Differentially expressed genes in antibody‐secreting cells during neoadjuvant chemotherapy are predictive of favourable prognoses in oesophageal cancer.^31^ In summary, B cells demonstrate potential as predictive biomarkers for chemotherapy response.

Although the associations between B cells and chemotherapy efficacy may involve B cell‐mediated immune functions, the underlying biological mechanisms remain unclear. Chemotherapy‐induced modulation of B cells could significantly impact tumour treatment outcomes. However, there is a lack of comprehensive reviews examining the interplay between chemotherapy and B cells. This review aims to elucidate the potential role of B cells in chemotherapy by summarising the effects of chemotherapy on B‐cell subtypes and their interactions within the TME. Our objective is to establish a theoretical foundation for harnessing the potential of B cells in chemotherapy and combination therapeutic regimens.

### 1.1 Normal B‐cell development and biology

**FIGURE 1:**
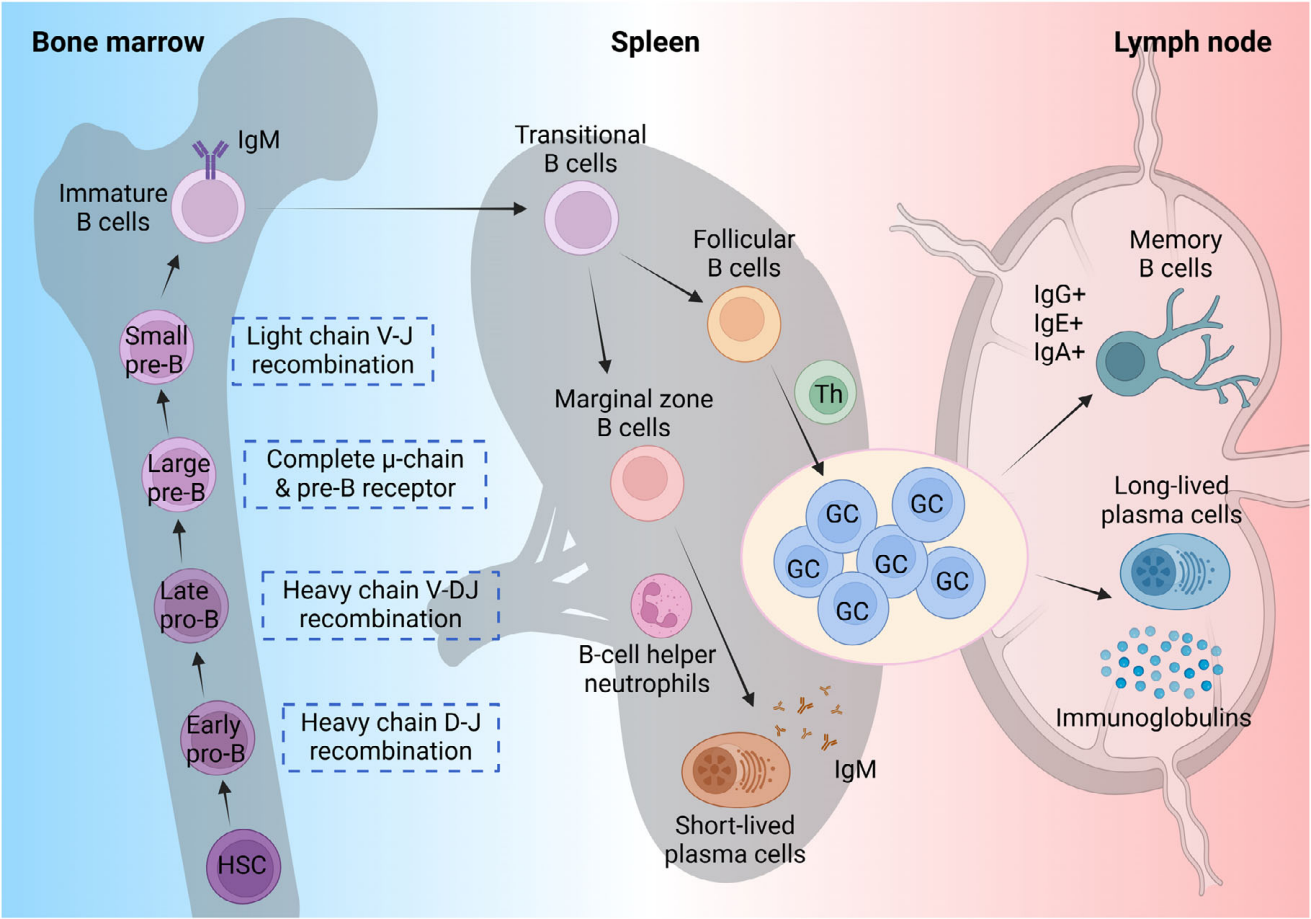
B‐cell biology and development. B cells originate from haematopoietic stem cells (HSCs) and undergo a multifaceted developmental process within the bone marrow. Lymphoid progenitor cells initially differentiate into Pro‐B cells, which progress through two distinct stages: early Pro‐B cells perform heavy chain D‐J recombination, whereas late Pro‐B cells undergo V‐DJ recombination. Subsequently, Pro‐B cells differentiate into Pre‐B cells, which are categorised into two types: large Pre‐B and small Pre‐B cells. Large Pre‐B cells possess the ability to synthesise a complete μ‐chain and express the Pre‐B receptor, while small Pre‐B cells commence V‐J recombination of the light chain but are unable to form a functional BCR. Immature B cells have completed light chain V‐J recombination and express surface IgM. Upon entering the spleen as transitional B cells, they mature into either marginal zone B cells or follicular B cells. Exposure to antigens and the influence of B‐cell helper neutrophils stimulate marginal zone B cells to differentiate into short‐lived plasma cells, which secrete IgM. Follicular B cells interact with helper T cells (Th cells) and are activated by Th‐cell‐derived cytokines, leading to the formation of germinal centres (GCs). Following a series of mutations, selection, and expansion phases, follicular B cells differentiate into memory B cells expressing surface IgG, IgE, or IgA, and long‐lived plasma cells secreting class‐switched immunoglobulins.

B cells originate from haematopoietic stem cells (HSCs) and undergo a complex developmental process within the bone marrow (Figure 1). Lymphoid progenitor cells first differentiate into Pro‐B cells, which express the Igα/Igβ heterodimer, a key hallmark of B cells. Pro‐B cells progress through two stages: early Pro‐B cells undergo heavy chain D‐J gene segment recombination, while late Pro‐B cells undergo V‐DJ gene segment recombination. When Pro‐B cells begin to express the immunoglobulin heavy chain, they differentiate into Pre‐B cells, which are classified into two types: large Pre‐B cells and small Pre‐B cells. Large Pre‐B cells synthesise a complete μ heavy chain and express the Pre‐B‐cell receptor, whereas small pre‐B cells initiate light chain V‐J gene segment recombination but do not produce a functional B‐cell receptor (BCR). Immature B cells are those that have completed light chain V‐J gene segment recombination and express surface IgM.^32^, ^33^ A significant proportion of these immature B cells are autoreactive and must be eliminated through negative selection processes. B cells that survive these negative selection processes express surface IgD and differentiate into mature B cells. This intricate series of developmental stages ensures that the B cells of the immune system are functional and self‐tolerant.^2^, ^34^, ^35^, ^36^, ^37^, ^38^

Immature B cells enter the spleen as transitional B cells and subsequently develop into either marginal zone B(MZ‐B) cells or FO‐B cells for further maturation (Figure 1). Naive B cells are defined as B cells circulating in the blood and lymph that have not encountered external antigens. Upon antigen exposure and with the assistance of B‐cell helper neutrophils, MZ‐B cells differentiate into short‐lived PCs that secrete IgM.^32^, ^33^ FO‐B cells bind to helper T (Th) cells and are activated by Th cell‐derived cytokines, ultimately leading to the formation of germinal centres (GCs). B cells are activated through contacts with antigen‐presenting cells (APCs), such as dendritic cells(DCs) and macrophages, in a T‐independent(TI) or T‐dependent(TD) manner.^2^, ^39^, ^40^ Following a series of mutations, selection and expansion phases, FO‐B cells differentiate into either MBCs expressing surface IgE, IgG, or IgA, or long‐lived PCs that secrete class‐switched immunoglobulins.

PCs, characterised by high expression levels of BLIMP1, IRF4 and XBP1^41^ and also known as antibody‐secreting cells, are the terminal effector cells in the B‐cell differentiation pathway. Upon antigen activation, MZ‐B cells differentiate into PCs via TI or TD pathways, synthesising and secreting various immunoglobulins to provide the initial rapid response to antigens.^42^, ^43^

MBCs, defined as canonical class‐switched CD27+ B cells,^44^ are produced in the germinal centre (GC) response during the TD immune response and account for about 40% of the total number of adult B cells.^45^ When stimulated by the same antigen for the second time, MBCs can rapidly proliferate and differentiate into PCs to produce antibodies more quickly and with greater strength, thereby developing protective immunity against recurrent infectious agents.^42^, ^45^

Bregs are a crucial class of immunosuppressive cells with diverse phenotypes, but there is no clear developmental relationship between Breg subpopulations. Bregs can produce IL‐10, IL‐35, and TGF‐β, which inhibit the growth of effector T cells through direct or indirect mechanisms. Furthermore, Breg cells promote the development of immunosuppressive T cells, Foxp3+ T cells, and regulatory T cells (Tregs).^46^

### 1.2 Key components of TME

Tumours arise from the continuous interaction and adaptation between tumour cells and the surrounding cells and stroma. The TME, comprising various stromal cells, immune cells, and ECM, plays a significant role in the pathophysiology of cancer. The primary stromal components and immune cells of the TME that are covered in this review are described below.

#### 1.2.1 Immune cells

Normal macrophages play essential roles in detecting, phagocytosing, and eliminating harmful microbes, such as bacteria, apoptotic cells, and metabolic waste, while also contributing to tissue homeostasis and immune defence against infections. Tumour‐associated macrophages (TAMs), as the most abundant innate immune cells in the TME, display both pro‐tumourigenic and tumour‐suppressive properties. Anti‐tumourigenic TAMs exhibit characteristics similar to APCs, as they express high levels of MHC II, demonstrate phagocytosis and tumour‐killing abilities and secrete pro‐inflammatory cytokines to promote and activate adaptive immune cells.^47^ In comparison, tumourigenic TAMs are immunosuppressive, exhibiting low MHC II expression and the presence of inhibitory molecules such as B7‐H4, VISTA, Tim3, PD‐1 and PD‐L1. These TAMs hinder antitumour responses by inhibiting phagocytosis, regulating angiogenesis, and suppressing the functions of immune cells, including DCs and T cells, thereby promoting tumour growth and metastasis.^48^, ^49^, ^50^, ^51^, ^52^, ^53^, ^54^

Natural killer (NK) cells are innate lymphocytes that develop from CD34+ lymphoid progenitor cells in the bone marrow.^55^ The function of NK cells is regulated by inhibitory and activating receptors, which detect tumour cells through various complementary mechanisms. Inhibitory receptors, such as NKG2A and inhibitory killer cell immunoglobulin‐like receptors (KIRs), can suppress the cytotoxic activity of NK cells.^56^ The upregulation of HLA‐E in cancer cells suppresses NK cell activation through NKG2A.^57^ The interaction of NK cells with HLA class I molecules regulates their overall function by modulating the amount of releasable granzyme B, which is essential for cytotoxic activity.^58^ Conversely, upon activation of their receptors, NK cells can induce target cell lysis through antibody‐dependent cellular cytotoxicity (ADCC) by releasing granzymes, granulysins and perforins.^59^, ^60^ NK cells can also initiate apoptosis in target cells by expressing FasL and TRAIL, which engage death receptors.^56^ NK cells can recruit and activate DCs, macrophages and T cells by secreting chemokines and cytokines, thereby promoting antitumour immunity.^61^, ^62^, ^63^, ^64^, ^65^

T cells, the primary adaptive immune cells, can be classified into CD8+ and CD4+ T cells based on their surface markers. Various chemokines and cytokines drive cDC1s to tumour tissues, where they internalise and process tumour antigens onto HLA‐I and HLA‐II, which are subsequently presented to CD8+ and CD4+ T cells. Naive CD4+ T cells are activated first, followed by CD8+ T cells through CD40‐CD40L signalling.^66^, ^67^ CD8+ T cells can be attracted by cDC1s producing chemokines CXCL9 and CXCL10, and are activated by IL‐12. CD8+ T cells exert their antitumour effects primarily through potent cytotoxicity, which can be mediated by TCR‐specific recognition of MHC peptide complexes expressed by cancer cells, as well as cancer cell killing via apoptosis mediated by granzymes and perforin or FasL‐Fas‐mediated cell death.^67^ CD4+ T helper cells support antitumour functions by assisting CD8+ T cells, B cells, and NK cells.^68^, ^69^, ^70^ They also directly kill cancer cells by producing IFN‐γ and TNF‐α, which induce the expression of perforin and granzyme.^71^, ^72^ In contrast, Th2 subtypes secrete anti‐inflammatory mediators that exert protumourigenic effects. However, Tregs, a highly immunosuppressive fraction of CD4+ T cells, have been shown to suppress tumour‐specific immune responses by reducing the infiltration and antitumour activity of CD8+ T cells and macrophages.^68^

#### 1.2.2 Stromal cells and stroma

Cancer‐associated fibroblasts (CAFs) are crucial components of the tumour stroma and can be categorised into three functionally distinct subtypes: antigen‐presenting CAFs, myofibroblasts CAFs, and inflammatory CAFs, all of which demonstrate remarkable plasticity.^73^ The functional activity of CAFs is modulated by various factors, such as inflammatory mediators, alterations in ECM composition, and metabolic changes, which collectively enable malignancies to evade immune regulation through multiple mechanisms.^73^, ^74^ For instance, CAFs create and remodel the ECM, establishing a physical barrier that directly modulates cancer cell signalling and behaviour, hinders immune cell recruitment and activation, and diminishes drug penetration into tumours.^73^ Moreover, CAFs secrete cytokines that suppress the recruitment and activation of effector T cells, facilitate the accumulation of Tregs, and foster an immunosuppressive microenvironment.^75^, ^76^, ^77^ Additionally, CAFs can directly enhance cancer cell proliferation and promote tumour formation.^73^

Angiogenesis provides essential oxygen and nutrients to support tumour growth and development. Carcinogenesis‐associated angiogenesis involves extensive interactions among multiple cell types, including cancer cells, tumour‐associated myeloid cells, endothelial cells, and CAFs, predominantly triggered by hypoxia.^73^ Growing evidence indicates that angiogenesis within the TME facilitates tumour immune evasion and immunosuppression by erecting barriers to immune cell infiltration, upregulating diverse immune checkpoint molecules, selectively eliminating effector T cells, and fostering immunosuppressive conditions.^73^

This review will further explore the impact of chemotherapy on the function of B‐cell subtypes and the modulated role of B cells within the TME. Furthermore, this review emphasises the tremendous potential of B‐cell targeting approaches in chemotherapy and combination therapy, striving to establish a theoretical foundation for B‐cell‐related cancer treatment.

## 2 CHEMOTHERAPY AFFECTS THE NUMBER AND FUNCTION OF B‐CELL AND THEIR SUBTYPES

**FIGURE 2:**
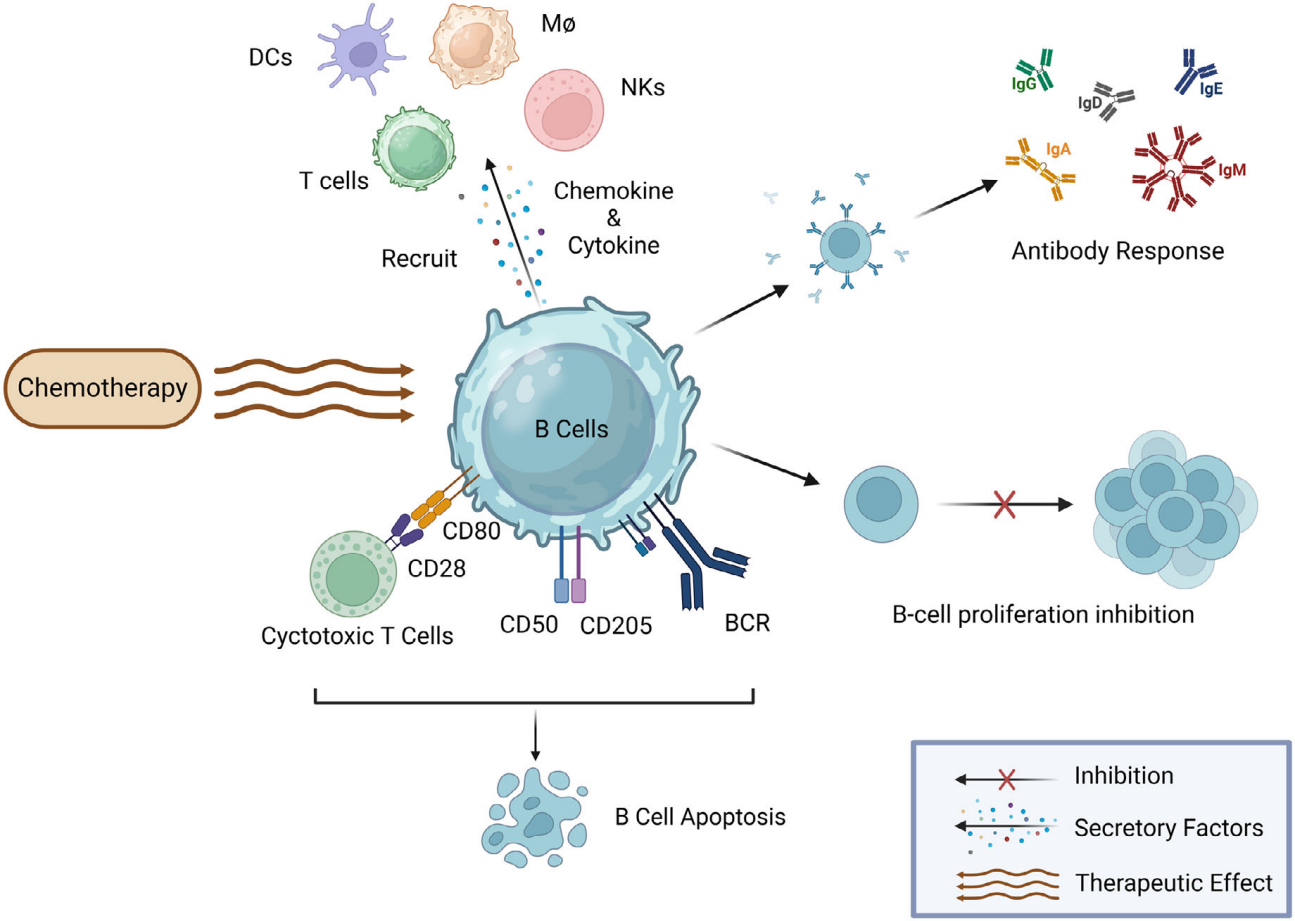
Effects of chemotherapy on B cells. Chemotherapy can inhibit B‐cell proliferation to varying degrees. B cells retain the ability to mount effective antibody responses following chemotherapy. Chemotherapy may stimulate B cells to secrete chemokines and cytokines, which recruit macrophages, dendritic cells, T cells and natural killer cells, indicating potential immunostimulatory effects. Chemotherapy‐induced alterations in cell surface proteins can influence B‐cell survival and apoptosis. BCR‐associated proteins can trigger apoptotic signalling pathways. Upregulation of the costimulatory molecule CD80 may facilitate the involvement of cytotoxic T cells in B‐cell death. Elevated levels of CD205 may contribute to the in vivo clearance of drug‐damaged cells.

Chemotherapy disrupts the balance between B‐cell depletion and proliferation, as well as alters the distribution of B‐cell subtypes (Figure 2). Multiple studies have demonstrated that various chemotherapeutic modalities can lead to substantial depletion of B cells.^18^, ^26^, ^78^ Combined treatment with cyclophosphamide, adriamycin, vincristine and prednisolone suppressed B‐cell proliferation and differentiation.^79^ Furthermore, chemotherapy induces alterations in the distribution of B‐cell subsets, which will be elaborated upon in the following section.

Chemotherapy additionally modulates B‐cell secretion of factors that mediate immune activation (Figure 2). Stimulation with low‐dose PMA/ionomycin enhanced B‐cell secretion of cytokines and chemokines, resulting in the recruitment of macrophages, DCs, T cells, and NK cells, thus indicating immunostimulatory effects.^23^ Purine analogues can potentially trigger apoptosis by interfering with BCR signalling and costimulatory molecules.^80^ Compared to untreated controls, neoadjuvant chemotherapy upregulated B‐cell TNF and HLA‐DQA2 expression in lymph nodes.^31^

In summary, chemotherapy can inhibit B‐cell proliferation and alter subset distributions and functions, including cytokine secretion, receptor signalling, and costimulatory molecule expression. However, B cells residing in lymph nodes may exhibit relative resistance to chemotherapy, thereby preserving antibody production. Furthermore, chemotherapy has been observed to exert immunostimulatory effects, such as enhanced immune cell recruitment to the TME. These complex and selective effects of chemotherapy on B cells may significantly influence anti‐tumour immune responses. In the following sections, we will discuss the effects of chemotherapy on various B‐cell subpopulations (Table 1).

**TABLE 1:** Summary of the effects of chemotherapy on B cells.

Drug	Type of tumour	Source of B cells	Effect on B cells	References
Cyclophosphamide	Mammary carcinoma	Blood	The percentage of B cells and T cells in peripheral blood remained the same before and after chemotherapy	[78]
			Chemotherapy induces considerable depletion of B lymphocytes	
Cisplatin, methotrexate and cyclophosphamide	Animal models (Wistar rats)	Bone marrow	All three chemotherapy treatments can cause Pro‐/Pre‐B cells in the bone marrow to decrease and then slowly rebound	[25]
		Spleen lymph nodes	After chemotherapy, marginal zone and follicular B cells diminish over a long period of time	
Cyclophosphamide, adriamycin, vincristine and prednisolone	Animal models (Wistar rats)	Lymph nodes	Combined treatment suppressed B‐cell proliferation and differentiation	[79]
Anthracycline‐based chemotherapy; anthracyclines and taxanes	Breast cancer	Blood	After chemotherapy, B cells were significantly depleted, and the expression of CD27 by B cells was reduced	[26]
			Chemotherapy increased the percentage of naive B cells while decreasing that of memory B cells	
			Restoration of CD4+ T cells correlated with memory B‐cell recovery after chemotherapy	
Platinum‐based chemotherapy	High‐grade serous ovarian metastases	lymphoid structures in the stroma of HGSOC metastases	Secrete cytokines and chemokines to recruit macrophages, dendritic cells, T cells and NK cells for immune function	[23]
			Metastatic sites exhibited a lower proportion of naive B cells compared to peripheral blood, suggesting that platinum‐based chemotherapy drove the differentiation and selection of naive B cells into memory B cells	
			Class‐switched MBC percentage and CD86 expression increased compared to IgM+ memory and naïve B cells	
			The inhibitory receptor PD1 is upregulated in all MBC subsets	
			Intratumoural plasma cells produced IgG recognising tumour antigens, forming immune complexes that activated dendritic cells and anti‐tumour responses	
Neoadjuvant chemotherapy (unclear)	Oesophageal cancer	Tumour tissues	High expression of TNF and HLA‐DQA2 in B cells	[31]
			Upregulate activating genes, diminish BCR signalling inhibition, and increase activation markers such CD27, CD70 and AIM2	
			MBC‐ITGAX is activated, allowing for better antigen presentation	
			Antibodies generated by intratumoural antibody‐secreting cells are enhanced	
			Chemotherapy‐activated T cells promote TIL‐B activation by stimulating CD40 molecules on B cells, NF‐κB pathways and antigen presentation	
		Lymph nodes (LNs)	The levels of expression of TNF and HLA‐DQA2 by B cells are increased in LNs receiving NACT compared to LNs not receiving NACT	
			Antibody‐secreting cells account for the largest proportion of metastatic LNs treated with NACT	
Chemotherapy (Cht) and iodine Cht: 5‐fluorouracil/epirubicin /cyclophosphamide or taxotere/epirubicin	Breast cancer	Tumour tissues	Activate both naive and memory B cells; the former can promote Th1 polarisation, while the latter rapidly create antibody responses to effectively limit tumour growth	[84]
Cyclophosphamide	Animal models (Wistar rats)	Spleen	Following cyclophosphamide treatment, follicular B cells and marginal zone B cells experienced a prolonged reduction, with marginal zone B cells being particularly affected	[88]
			Marginal zone B cells were significantly reduced after Cyclophosphamide treatment and have not yet fully recovered, but a normal immune response to TI‐2 antigen was still observed, implying that the presence of a small number of marginal zone B cells was sufficient to cause an increase in antibody titres	
Methotrexate	Animal models (Wistar rats)	Spleen	Methotrexate can inhibit B‐cell function, making B cells unable to function properly	[88]
Gemcitabine	Pancreatic ductal adenocarcinoma	Blood	Increased IgG response to tumour‐associated antigens	[90]
Doxorubicin	Urothelial urinary bladder cancer	Blood	Doxorubicin caused B cells to highly express CD86, contributing to T‐cell activation, while reducing TNF‐α secretion that can mediate Treg blockade	[93]
Platinum‐based chemotherapy	Head and neck squamous cell carcinoma	Blood	Platinum‐based treatment reduces not only the frequency of Breg, but also its ability to create immunosuppressive adenosine	[94]
			Methotrexate treatment improves the efficacy of anti‐inflammatory therapy by boosting Breg's ADO‐producing function and ability to block CD4+ T cells	
XELOX regimen (capecitabine plus oxaliplatin)	Gastric cancer	Blood	Bregs can demonstrate a high frequency of apoptosis following treatment in a dose‐dependent way, implying that lowering Bregs may improve patient immunological function and accomplish the intended chemotherapeutic effect	[95]
Doxorubicin, cyclophosphamide and paclitaxel or docetaxel and cyclophosphamide	Breast cancer	Tumour tissues	The levels of ICOSL and CR2 in tumour‐infiltrating B cells were significantly increased after chemotherapy, while IL‐10 expression was significantly inhibited	[27]
			There was no significant change in the percentage of total B cells in the tumour	
			ICOSL generated in tumour‐infiltrating B cells conferred an antitumour T‐cell immune response and increased chemotherapeutic efficacy	
		Blood	ICOSL+ B cells in the blood increased, while total B cells remained the same	
Gemcitabine plus cisplatin chemotherapy	Nasopharyngeal carcinoma	Tumour tissues	Chemotherapy activates innate B cells to amplify Tfh and Th1 cells by ICOSL‐ICOS and enhances cytotoxic T‐cell function	[98]
Platinum‐based chemotherapy	Muscle‐invasive bladder cancer	Tumour tissues	B cells may activate CD4+ T cells as antigen presenting cells to improve outcomes with chemotherapy	[99]
Neoadjuvant chemotherapy (Unclear)	Pancreatic ductal adenocarcinoma	Tumour tissues	Chemotherapy reversed PD‐L1/PD‐1 suppression of Tfh cells, permitting CXCL13‐mediated B‐cell recruitment and IL‐21‐driven plasma cell development, hence increasing anti‐tumour immunity	[101]
Oxaliplatin or cyclophosphamide	MC38 tumour‐bearing mice	Spleen	CD19+ EVs formed from B cells are rich in CD39 and CD73 vesicle fusion proteins, which can hydrolyse ATP produced by chemotherapy‐induced tumour cells to adenosine, limit CD8+ T‐cell proliferation and anti‐tumour actions, and therefore reduce the efficacy of chemotherapy	[102]
Low‐dose oxaliplatin	Prostate cancer	Tumour tissues	Low‐dose oxaliplatin improves survival in TRAMP mice in a manner dependent on CTL and by inhibiting B cells	[105]
			B‐cell immune reduction can increase oxaliplatin‐induced tumour regression, which is CTL‐dependent	
Chemotherapy (unclear)	Haematologic malignancies	Blood	A relative increase in the proportion of naïve B cells can be observed during the chemotherapy recovery phase	[81, 82]
Purine analogs	Haematological malignancies	Blood	Chemotherapy induces apoptosis by affecting B‐cell receptor (BCR) signalling and costimulatory molecules	[80]
Cytotoxic chemotherapy (unclear)	Haematological malignancies	Blood	One year after discontinuing chemotherapy, transitional B cells rebounded to slightly above normal levels	[86]
			Chemotherapy gradually reduced the number of memory B cells, which did not restore to normal levels even a year later	
Cyclophosphamide	Haematological malignancies	Blood	The reduction in IgE caused by chemotherapy was significantly less pronounced than the decrease in B‐cell count, suggesting that a subset of PCs could survive for several weeks	[89]
Cytotoxic chemotherapy (unclear)	Haematological malignancies	Blood	The recovery of B‐cell subsets was delayed compared to T‐cell recovery	[85]
			Compared with the healthy, the proportion of transitional B cells increased after chemotherapy in AML patients, while the proportion of memory B cells decreased	
			Approximately 6 months following chemotherapy, transitional and naive B cells gradually return to normal. However, memory and effector B cells do not entirely recover	
ALL: Dutch Childhood Oncology ALL‐9 protocol or Interfant 99 trial; AML: MRC AML12 trial; NHL: NHL 94 trial or NHL–BFM trial; HL: GPOH‐HD‐95 trial	Haematological malignancies	Blood	Newly generated transitional and naive B cells recover quickly within months, while memory B cells recover slowly and incompletely even after 5 years of chemotherapy	[87]
Rituximab and chemotherapy (THP–COP–BLM therapy)	Haematological malignancies	Blood	Combination therapy can induce sustained B‐cell differentiation arrest and apoptosis	[83]
			After THP‐COP‐BLM therapy, IgD+CD27‐ naive B cells naive B cells predominate in B cells	

Abbreviations: ALL, acute lymphoblastic leukaemia; AML, acute myeloid leukaemia; HL, Hodgkin lymphoma; NHL, non‐Hodgkin lymphoma; THP‐COP‐BLM therapy, consisting of pirarubicin, cyclophosphamide, vincristine, bleomycin, prednisolone, procarbazine.

### 2.1 Incompletely mature B cells

#### 2.1.1 Naive B cells

**FIGURE 3:**
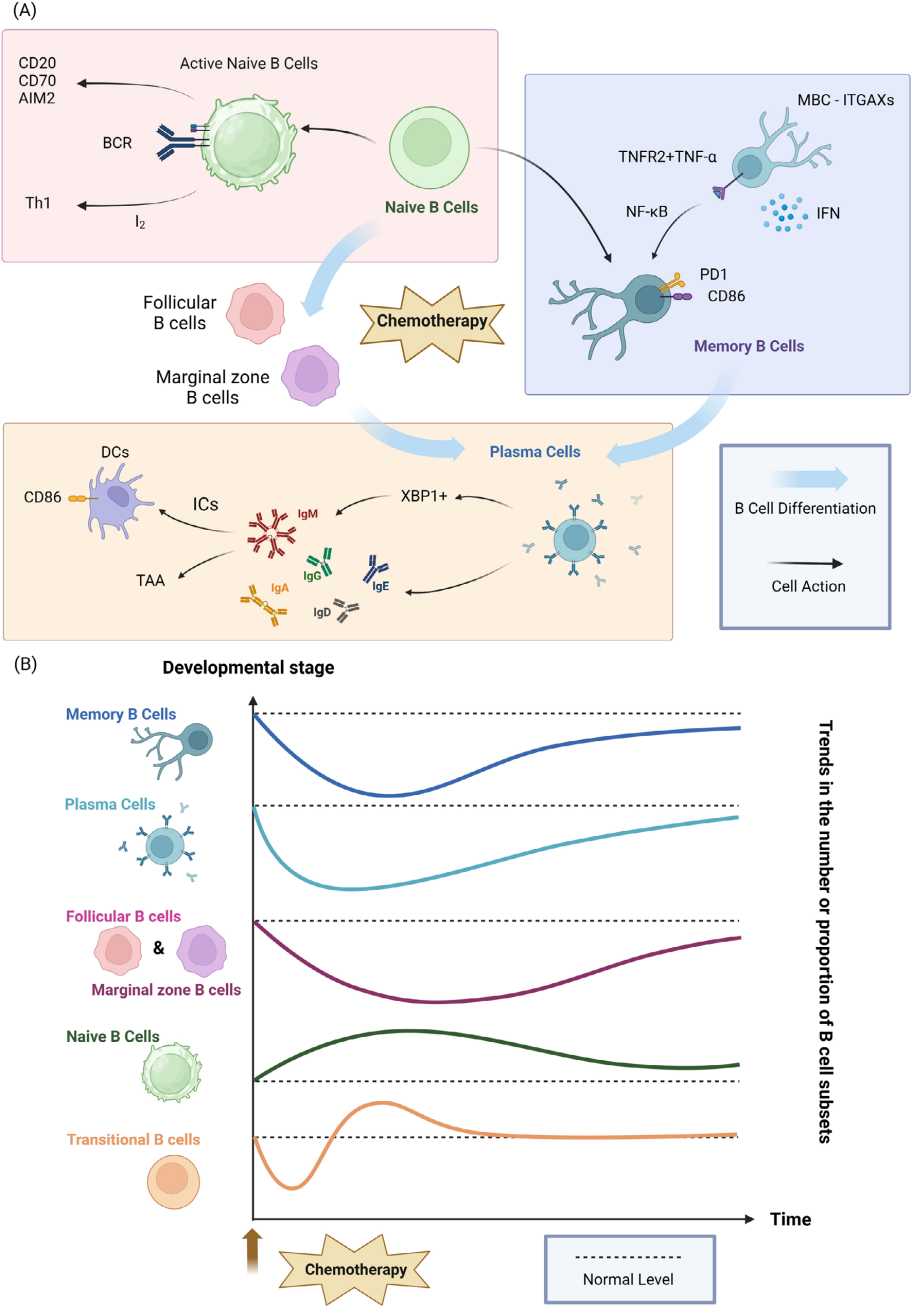
The impact of chemotherapy on various B‐cell subsets. (A) Changes in function of B‐cell subsets. (1) Naive B cells: Chemotherapy has been shown to upregulate activation genes and markers such as CD27, CD70, and AIM2 while simultaneously downregulating inhibitory receptors, resulting in attenuated BCR signalling inhibition. Furthermore, chemotherapy may promote the polarisation of naive B cells towards a Th1 phenotype in the presence of iodine. (2) Plasma cells: Chemotherapy has been demonstrated to enhance XBP1 expression and IgG production, facilitating the recognition of tumour antigens and the formation of immune complexes. Moreover, chemotherapy can stimulate the expression of the CD86 costimulatory molecule on dendritic cells in vitro and augment IgG responses directed against tumour‐associated antigens. (3) Memory B cells: Chemotherapy induces the upregulation of the inhibitory receptor PD1 on all memory B‐cell subsets. Additionally, the costimulatory molecule CD86 exhibits increased expression on certain memory B‐cell subsets following chemotherapy. Furthermore, chemotherapy induces the upregulation of TNFR2 on the MBC‐ITGAX subset, potentially enabling the binding of TNFα and subsequent activation of NF‐κB signalling. Moreover, the MBC‐ITGAX subset may also augment antigen presentation following stimulation with type I interferons. (B) Trends in the number or proportion of B‐cell subsets. (1) Transitional B cells: Transitional B cells recover rapidly after chemotherapy, initially exceeding normal levels and then falling back to normal levels; (2) Naive B cells: chemotherapy causes an increase in the proportion of naïve B cells; (3) Follicular B cells & Marginal zone B cells: After chemotherapy, follicular and marginal zone B cells drop and recover later than other subsets, particularly marginal zone B cells; (2) Plasma cells: Chemotherapy decreases the number of plasma cells; however, it inhibits immunoglobulins far less than it reduces the number of B cells; (3) Memory B cells: Chemotherapy can gradually lower the proportion of memory B cells. Memory cells recover slowly over time, but not completely.

Chemotherapy elevates the proportion of naive B cells, facilitating their activation and differentiation into MBCs through clonal selection (Figure 3). Moreover, it attenuates the inhibition of BCR signalling. In breast cancer patients, chemotherapy increased the percentage of naive B cells while decreasing that of MBCs.^26^ Analogous increases in naive B cells were observed in haematologic malignancies,^81^, ^82^ implying that repopulation relies on naive B cells from the bone marrow. This finding was corroborated by the predominance of IgD+CD27‐naive B cells following rituximab and chemotherapy (THP–COP–BLM therapy).^83^ In ovarian cancer, metastatic sites exhibited a lower proportion of naive B cells compared to peripheral blood, suggesting that platinum‐based chemotherapy drove the differentiation and selection of naive B cells into MBCs.^23^ The combination of chemotherapy (Cht+I2) and iodine elevated the number of naive B cells and facilitated Th1 polarisation.^84^ In postchemotherapy naive B cells, activation genes and markers such as CD27, CD70 and AIM2 were upregulated, while inhibition of BCR signalling was downregulated.^31^

#### 2.1.2 Transitional B cells

Studies have shown that transitional B cells recover rapidly after chemotherapy, initially exceeding and then decreasing to normal levels, whereas memory B‐cell recovery is slower (Figure 3).^26^, ^83^, ^85^, ^86^ This finding may explain the diminished immune function observed postchemotherapy. Transitional B cells were severely depleted but rebounded within months, in contrast to the incomplete recovery of MBCss even years later.^83^, ^85^ The early recovery of transitional B cells was most pronounced in paediatric patients.^87^ In patients with leukaemia, transitional B‐cell frequencies increased and memory B‐cell frequencies decreased after chemotherapy compared to healthy controls.^86^ The impaired antibody responses to influenza vaccination postchemotherapy may be attributed to the lack of mature and transitional B cells.^87^

#### 2.1.3 Others

All three cell inhibitors, cisplatin, methotrexate and cyclophosphamide, caused severe myelosuppression; however, cyclophosphamide and methotrexate only slightly reduced spleen cell populations.^25^ Doxorubicin therapy can cause dose‐dependent spleen dysplasia; however, it does not impact cell population distribution.^18^ Following cyclophosphamide treatment, FO‐B cells and MZ‐B cells experienced a prolonged reduction, with MZ‐B cells being particularly affected.^88^ These two cell subsets exhibited delayed recovery compared to other cell subsets.^25^ Despite the significant reduction and incomplete recovery of MZ‐B cells following cyclophosphamide treatment, a normal immune response to the T‐independent type 2 (TI‐2) antigen was still observed, suggesting that the presence of a small number of MZ‐B cells was sufficient to elicit an increase in antibody titres.^88^ Treatment with cyclophosphamide resulted in only mild myelosuppression and had minimal to no impact on the number of B cells in the spleen.^88^ Although the spleen treated with methotrexate exhibits a largely unchanged cell count, it is probable that B cells are not functioning optimally, as methotrexate is not directly cytotoxic but rather inhibits B‐cell function.^88^

### 2.2 Completely mature B cells

#### 2.2.1 Plasma cells (PCs)

The suppression of immunoglobulin production by chemotherapy is considerably less pronounced than the decrease in B‐cell counts (Figure 3). In leukaemia patients, the recovery of B‐cell subsets was delayed compared to T‐cell recovery, possibly due to the inherent susceptibility of effector B cells to chemotherapy, leaving insufficient time for complete population restoration.^85^ Postchemotherapy decreases in allergen‐specific IgE were significantly less pronounced than the decline in B‐cell counts, suggesting that a portion of PCs can survive for several weeks.^89^ Although lymphocyte, T‐cell, and B‐cell counts persisted at low levels one year after chemotherapy, functional markers predominantly recovered within 3 months.^17^

In addition to its suppressive effects, chemotherapy has the potential to stimulate immunoglobulin production and activate the immune system (Figure 3). In ovarian cancer patients, CD38++CD19+/‐CD20‐CD27+ PCs and IgG, particularly IgG3, were detected following platinum‐based chemotherapy treatment.^23^ IgG primarily accumulated in the stroma, as platinum‐based chemotherapy‐induced cell death released antigens, facilitating the accumulation of antigen‐specific IgG.^23^ Intratumoural PCs secreted IgG that recognised tumour antigens, leading to the formation of immune complexes, which activated DCs and triggered anti‐tumour responses.^23^ In oesophageal cancer, antibody secreting genes were enriched after neoadjuvant chemotherapy, increasing antibody production by intratumoural antibody‐secreting cells.^31^ Similarly, in pancreatic cancer, chemotherapy increased IgG responses against tumour‐associated antigens.^90^

#### 2.2.2 Memory B cells

Chemotherapy can gradually decrease the proportion of MBCs without full recovery over time (Figure 3). In haematologic cancers, MBC subsets declined after chemotherapy,^13^, ^15^ especially IgM+ MBCs in medium‐risk patients.^15^ IgA+ and IgM‐only MBCs recovered faster than IgG+ and IgM+ MBCs, but no populations fully recovered even after the completion of chemotherapy.^13^, ^15^ Rituximabin combination with chemotherapy (THP‐COP‐BLM therapy) may induce a sustained blockade of MBC differentiation and apoptosis, resulting in a marked reduction of mature IgD‐CD27+ class‐switched MBCs.^83^ In breast cancer, the percentage of MBCs significantly dropped after 3 months of chemotherapy and remained below baseline levels thereafter.^26^

Chemotherapy also induces memory B‐cell (MBC) activation and alters the expression of costimulatory molecules (Figure 3). In ovarian cancer, most tumour‐infiltrating B cells displayed classical or atypical memory phenotypes. Neoadjuvant chemotherapy increased the percentages of class‐switched MBCs and upregulated CD86 expression on class‐switched MBCs compared to IgM+ memory and naive B cells. All MBC subsets upregulated the expression of the inhibitory receptor PD1.^23^ In oesophageal cancer, chemotherapy enhanced the activation of the MBC‐ITGAX subtype through TNFα/NF‐κB and IL2/STAT5 signalling pathways. The upregulation of TNFR2 may trigger NF‐κB signalling, leading to MBC activation. The MBC‐ITGAX subtype may also enhance antigen presentation following type I interferon stimulation.^31^

In summary, chemotherapy depletes MBCs, and the recovery is often incomplete. However, chemotherapy also activates MBCs and modulates the expression of costimulatory and inhibitory molecules, which likely impacts memory responses.

### 2.3 Regulatory B cells (Bregs)

Bregs are known to play an inhibitory role in the immune response against tumours.^91^, ^92^ Chemotherapy has been shown to inhibit interleukin‐10 (IL‐10) production by Bregs and induce increased apoptosis in Bregs, potentially enhancing anti‐tumour immunity and improving the efficacy of chemotherapy (Figure 3).

It has been reported that IL‐10 production within B cells treated with doxorubicin is inhibited, leading to a reduction in the suppressive effect of B cells.^93^ The tumour escape mechanism is facilitated by the presence of immunosuppressive adenosine (ADO), which has been demonstrated in vitro and in two separate cohorts of head and neck squamous cell carcinoma (HNSCC) patients. Platinum‐based therapy has been shown to reduce not only the frequency of Bregs but also their ability to produce immunosuppressive ADO.^94^ Compared with other immune cells, Bregs can exhibit a higher frequency of apoptosis after chemotherapy with the XELOX regimen (capecitabine plus oxaliplatin). Furthermore, Bregs can secrete the inhibitory cytokine IL‐10. Therefore, Jiao Yang et al. speculated that the reduction of Bregs may improve the immune function of patients and enhance the efficacy of chemotherapy.^95^

Multiple studies have shown a negative correlation between disease progression and the number and proportion of Bregs, which seems to confirm this hypothesis. In the early stages of gastric cancer treatment, the dynamic changes of peripheral blood Bregs are important for predicting the response to chemotherapy, and patients with decreased Bregs after XELOX chemotherapy exhibited longer PFS than those with increased Bregs.^96^ Furthermore, other studies have shown that a decrease in the proportion of peripheral blood Bregs is associated with a better prognosis in liver cancer patients treated with sorafenib in combination with chemotherapy.^97^

## 3 B‐CELL–T‐CELL INTERACTIONS UNDER CHEMOTHERAPY

**FIGURE 4:**
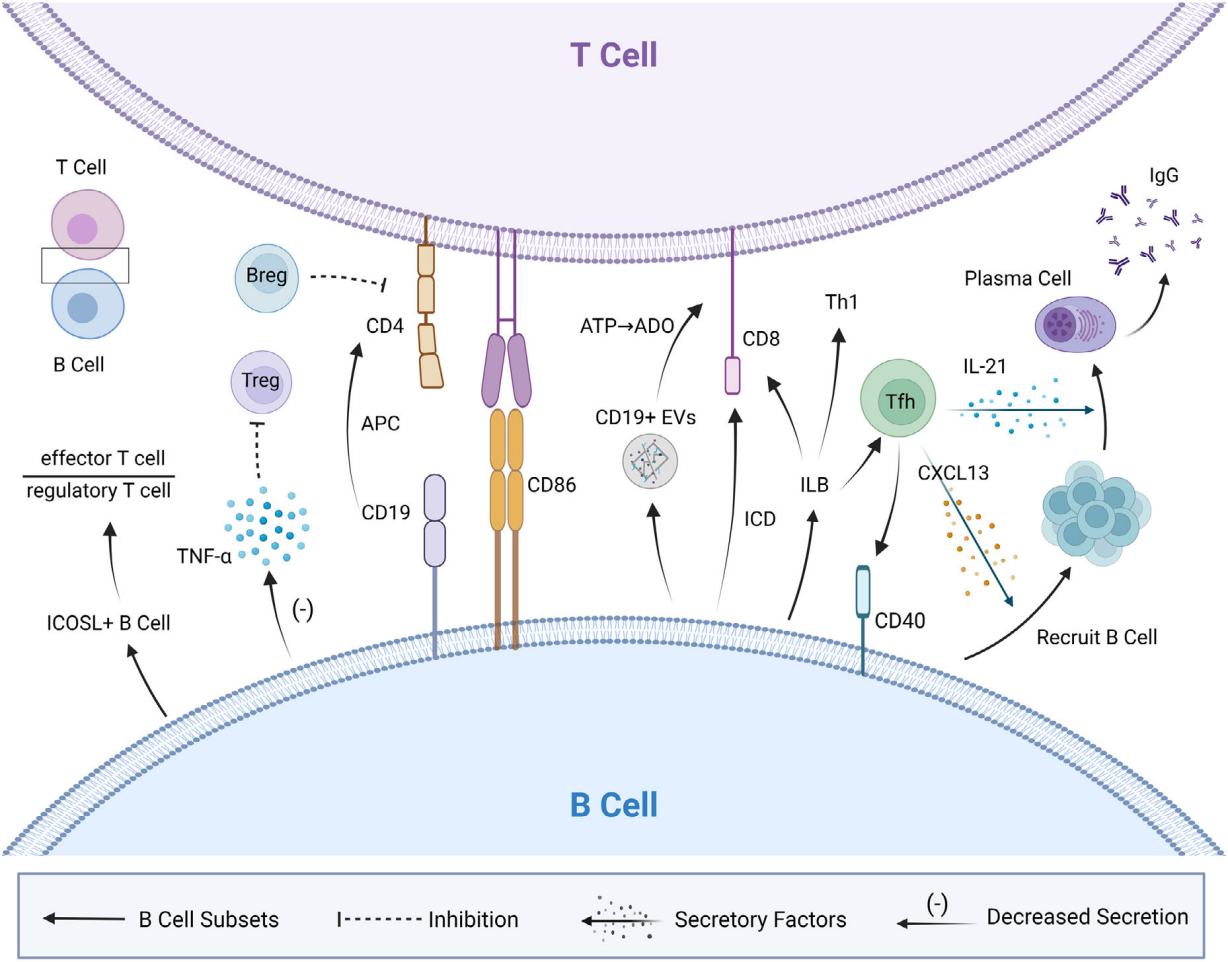
B‐cell and T‐cell interactions during chemotherapy. Chemotherapy induces the expression of CD86 on B cells, which activates T cells. CD19+ B cells can function as antigen‐presenting cells (APCs) to activate CD4+ T cells; however, methotrexate enhances the inhibitory effects of regulatory B cells on CD4+ T cells. Chemotherapy decreases the secretion of tumour necrosis factor‐alpha (TNF‐α) by B cells, potentially impeding the activation of regulatory T cells (Tregs). Extracellular vesicles derived from B cells generate immunosuppressive adenosine from ATP in chemotherapy‐treated tumour cells through the CD39/CD73 pathway, thereby hindering the function of CD8+ T cells. Conversely, B cells can promote the activation of cytotoxic T cells by inducing immunogenic cell death in tumour cells during chemotherapy. The emergence of ICOSL‐expressing B cells increases the ratio of effector T cells to regulatory T cells, thereby enhancing anti‐tumour immune responses. Chemotherapy activates innate‐like B cells, which expand follicular helper T (Tfh) cells and type 1 helper T (Th1) cells through the ICOSL‐ICOS pathway, ultimately boosting cytotoxic T‐cell function. Activated Tfh cells stimulate CD40 on B cells and may promote B‐cell activation through the NF‐κB pathway and enhanced antigen presentation. CXCL13 secreted by Tfh cells recruits B cells, while interleukin‐21 (IL‐21) promotes the differentiation of B cells into plasma cells, ultimately enhancing anti‐tumour immunity mediated by IgG subclasses.

Additionally, in the presence of chemotherapy, B cells can modulate T‐cell responses (Figure 4). Chemotherapy can induce B cells to activate T cells and inhibit Tregs, thereby promoting anti‐tumour immunity. Doxorubicin induced high expression of CD86 on B cells, contributing to T‐cell activation, while simultaneously reducing TNF‐α secretion, which can mediate Treg inhibition.^93^ In breast cancer, an increase in ICOSL+ B cells following neoadjuvant chemotherapy further enhanced anti‐tumour immunity and efficacy by elevating the ratio of effector T cells to Tregs.^27^ In nasopharyngeal carcinoma, gemcitabine plus cisplatin chemotherapy activated innate‐like B cells, leading to the expansion of type 1 T helper (Th1) cells and follicular helper T (Tfh) cells through ICOSL‐ICOS interaction, ultimately enhancing cytotoxic T‐cell function.^98^ In bladder cancer, CD19+ tumour‐infiltrating B cells may activate CD4+ T cells through antigen presentation, thereby improving outcomes with platinum‐based chemotherapy.^99^

Chemotherapy may also enhance anti‐tumour immunity by promoting T‐cell‐mediated activation of B cells (Figure 4). In oesophageal cancer, neoadjuvant chemotherapy activated T cells, including Tfh cells, which stimulate CD40 on B cells to promote their activation and anti‐tumour effects via NF‐κB signalling and antigen presentation.^31^ The restoration of CD4+ T cells correlated with the recovery of MBCs following chemotherapy in breast cancer.^26^ Synergistic increases in B cells and CD4+ T cells were associated with an improved response to neoadjuvant chemoimmunotherapy.^100^ In pancreatic cancer, neoadjuvant chemotherapy reversed the PD‐L1/PD‐1‐mediated inhibition of Tfh cells, enabling CXCL13‐mediated recruitment of B cells and IL‐21‐driven differentiation of PCs, ultimately enhancing anti‐tumour immunity.^101^

Additionally, B cells can modulate T‐cell responses, thereby impacting chemotherapy efficacy (Figure 4). HIF‐1α promotes the creation of CD19+ extracellular vesicles (EVs) in tumour B cells by increasing Rab27A expression. CD19+ EVs are rich in CD39 and CD73 vesicle fusion proteins, which can hydrolyse ATP generated by chemotherapy‐induced tumour cells into adenosine.^102^ Extracellular adenosine suppresses CD8+ T‐cell proliferation and antitumour activity, principally through A2A adenosine receptor (A2AR) signalling on T‐cell surfaces, thereby reducing chemotherapeutic efficacy.^102^, ^103^, ^104^ However, in a mouse model, B cells promoted cytotoxic T‐cell activation by inducing immunogenic cell death in tumour cells treated with low‐dose oxaliplatin chemotherapy.^105^ Methotrexate also enhanced the suppressive function of Bregs on CD4+ T cells.^94^

In summary, chemotherapy can modulate the complex interactions between T cells and B cells, leading to variable effects on anti‐tumour immunity.

## 4 TARGETING B CELLS AFFECTS CLINICAL OUTCOMES

### 4.1 Targeting B cells affects chemotherapy efficacy

Targeting B‐cell surface markers or signalling pathways has been shown to enhance chemotherapy efficacy, prevent immune evasion and suppress tumour growth (Table 2; Table 3). Studies have demonstrated that B‐cell‐deficient mice are resistant to squamous cell carcinoma growth, and B‐cell depletion using CD20 monoclonal antibodies prior to chemotherapy improves the efficacy of platinum and paclitaxel.^106^ The B and T lymphocyte attenuator (BTLA), a co‐inhibitory receptor, is highly expressed on B cells. In preclinical models, inhibition of BTLA or depletion of B cells in combination with chemotherapy results in enhanced immune activation and more potent antitumour effects compared to chemotherapy alone, suggesting potential clinical applications.^107^ Chemotherapy upregulates the expression of WNT16B in tumours through the activation of NF‐κB, which in turn attenuates the cytotoxic effects of chemotherapy by activating Wnt signalling. Therefore, targeting WNT16B may potentially enhance the efficacy of genotoxic therapy.^108^ Similarly, inhibition of upstream regulators such as NF‐κB may also potentiate the effects of chemotherapy.^109^ Hypoxic conditions upregulate the expression of Rab27a in B cells and promote the release of CD19+ extracellular vesicles, which generate immunosuppressive adenosine from treated tumours through the CD39/CD73 pathway, ultimately impairing the function of CD8+ T cells. Consequently, knockdown of Rab27a or hypoxia‐inducible factors in B cells may potentially enhance the response to chemotherapy.^102^

**TABLE 2:** Summary of clinical trials of B‐cell‐related targets in solid tumours.

Target	Drug	Combination agent	Phase	Tumour type	Clinical trial	Status
BTLA	TAB004	Toripalimab	Phase I	Advanced unresectable solid tumour; metastatic solid tumour	NCT04137900	Recruiting
	JS004	Toripalimab; Docetaxel; Pemetrexed; Cisplatin; Carboplatin; Paclitaxel; Etoposide	Phase Ib/II	Advanced lung cancer	NCT05664971	Recruiting
	HFB200603	Tislelizumab	Phase I	RCC; melanoma; NSCLC; GC; CRC	NCT05789069	Recruiting
hypoxia factor	Belzutifan (PT2977)	–	Phase I	Advanced solid tumours; solid tumour; KC; RCC; GBM	NCT02974738	Active, not recruiting
	Topotecan	Fluorine‐19‐Fluoroded Xyglucose	Phase I	Neoplasms	NCT00117013	Completed
	Intravenous EZN‐2968 (anti‐HIF‐1α LNA AS‐ODN)	–	Phase I	Carcinoma; lymphoma	NCT00466583	Completed
CD55 (DAF)	Gebasaxturev (V937‐013)	Pembrolizumab	Phase Ib/II	Neoplasm metastasis	NCT04521621	Terminated
Bruton's tyrosine kinase	Ibrutinib	Durvalumab	Phase Ib/II	NSCLC; BC; PC	NCT02403271	Completed
NF‐κB	PS‐3419 (VELCADE)	Temozolomide	Phase I/II	Brain and central nervous system tumours; melanoma; solid tumour	NCT00512798	Terminated
	ASTX660	–	Phase I/II	Solid tumours; lymphoma	NCT02503423	Active, not recruiting
AXL	Enapotamab vedotin (HuMax‐AXL‐ADC)	–	Phase I/II	OC; NSCLC; TC; CC; endometrial cancer; melanoma; sarcoma; solid tumours	NCT02988817	Completed
	SLC‐391	–	Phase I	Solid tumour	NCT03990454	Completed
	ADCT‐601	Gemcitabine	Phase I	Advanced solid tumours	NCT05389462	Recruiting
	XZB‐0004	–	Phase I	Advanced solid tumour; NSCLC	NCT05772455	Not yet recruiting
	CAB‐AXL‐ADC	PD‐1 inhibitor	Phase I/II	Undifferentiated pleomorphic sarcoma; myxofibrosarcoma	NCT03425279	Recruiting
	TP‐0903	–	Phase I	Advanced solid tumours; EGFR positive NSCLC; CRC; recurrent OC; BRAF‐mutated melanoma	NCT02729298	Completed
	RXDX‐106	–	Early Phase I	Advanced or metastatic solid tumours	NCT03454243	Terminated
	MGCD516	–	Phase I	Advanced cancer	NCT02219711	Completed
	INCB081776	INCMGA00012	Phase I	Advanced solid tumours	NCT03522142	Active, not recruiting
	Crizotinib	–	Phase II	Haematologic cancers; solid tumours; metastatic cancer	NCT02034981	Completed
	FC084CSA	–	Phase I	Advanced malignant solid tumours	NCT06231550	Recruiting
	BPI‐9016 M	–	Phase I	Solid tumours	NCT02478866	Completed
	PF‐07265807	Sasanlimab; Axitinib	Phase I	Neoplasm metastasis	NCT04458259	Active, not recruiting
	Q702	–	Phase I	Solid tumour; advanced cancer; metastatic cancer	NCT04648254	Recruiting
		Pembrolizumab	Phase Ib/II	Oesophageal cancer; GC; HCC; CC	NCT05438420	Recruiting
	MGCD265	–	Phase I	Advanced cancer	NCT00697632	Completed
CSF1R	ARRY‐382	Pembrolizumab	Phase Ib/II	Advanced solid tumours	NCT02880371	Terminated
	LY3022855	Durvalumab;Tremelimumab	Phase I	Solid tumour	NCT02718911	Completed
	Q702	–	Phase I	Solid tumour; advanced cancer; metastatic cancer	NCT04648254	Recruiting
		Pembrolizumab	Phase Ib/II	Oesophageal cancer; GC; HCC; CC	NCT05438420	Recruiting
	IMC‐CS4	–	Phase I	Neoplasms	NCT01346358	Completed
	BLZ945	PDR001	Phase I/II	Advanced solid tumours	NCT02829723	Terminated
	Axatilimab (SNDX‐6352)	Durvalumab	Phase I	Solid tumour; metastatic tumour; locally advanced malignant neoplasm; unresectable malignant neoplasm	NCT03238027	Completed
		Durvalumab	Phase II	Unresectable intrahepatic cholangiocarcinoma	NCT04301778	Completed
	PLX3397	Pembrolizumab	Phase I/IIa	Melanoma; NSCLC; squamous cell carcinoma of the head and neck; gastrointestinal stromal tumour; OC	NCT02452424	Terminated
	Pexidartinib	–	Phase III	Pigmented villonodular; giant cell tumours of the tendon sheath; synovitis; tenosynovial giant cell tumour	NCT02371369	Completed
	elzovantinib (TPX‐0022)	–	Phase I/II	Advanced solid tumour; metastatic solid tumours; MET gene alterations	NCT03993873	Active, not recruiting

Abbreviations: BC, breast cancer; CC, cervical cancer; CRC, colorectal cancer; GBM, glioblastoma; GC, gastric cancer; HCC, hepatocellular cancer; KC, kidney cancer; NSCLC, nonsmall cell lung cancer; OC, ovarian cancer; PC, pancreatic cancer; RCC, renal cell carcinoma; TC, thyroid cancer.

**TABLE 3:** Summary of untapped B‐cell‐related targets.

Target	Overview of the target	Publication time	Conclusions of the study	DOI
WNT16B	Chemotherapy‐induced DNA damage can increase WNT16B protein production in prostate fibroblasts, which is regulated by NF‐κB in B cells. WNT16B activates the classical Wnt program in tumour prostate epithelial cells in a paracrine manner, and Wnt signals can acquire mesenchymal cell properties via epithelial to mesenchymal transition, influencing epithelial cell migration and invasion behaviour, weakening the effect of cytotoxic chemotherapy in vivo, and promoting tumour cell survival and disease progression.	March 19, 2019	Chemotherapy‐induced DNA damage secretory program‐associated tumour microenvironment damage, such as WNT16B, can promote prostate cancer treatment resistance; thus, targeting WNT16B is an appealing target to improve response to more general genotoxic treatments.	https://doi.org/10.1038/nm.2890
Rab27a	Hypoxia enhances Rab27a expression in tumour B cells, causes greater release of Cd19+ Ev and hydrolyses ATP to immunosuppressive adenosine via CD39/CD73, weakening CD8+ T cells and reducing the efficacy of chemotherapy.	June 11, 2013	IEBVs‐Rab27a siRNA can diminish B‐cell‐derived extracellular vesicles, cause immunosuppression, boost Cd8+ T‐cell response following chemotherapy, and increase chemotherapy efficacy.	https://doi.org/10.1016/j.immuni.2019.01.010

### 4.2 Targeting TME affects chemotherapy efficacy

Targeting tumour cells or macrophages influences B‐cell function, enhancing anti‐tumour immunity and suppressing tumour growth, thereby improving chemotherapy efficacy (Table 1). Aberrant expression of CD55 on tumour cells impairs chemotherapy efficacy by inhibiting the induction of ICOSL+ B cells. Targeting CD55 could potentially restore complement‐mediated immune activation.^27^ Antibody‐drug conjugates, such as anti‐AXL‐MMAE, selectively eliminate tumour cells while sparing healthy tissue, transforming the microenvironment from immunosuppressive to anticancer by synergistically enhancing B and other immune cells, resulting in superior regression of large tumours compared to chemotherapy alone.^110^ Targeting macrophages in combination with chemotherapy expands activated B cells, which serve as major APCs that interact with T cells to mediate anti‐tumour immunity.^111^ In the context of pancreatic cancer, inhibition of Bruton's tyrosine kinase (BTK) disrupted B‐cell–macrophage interactions, restoring T‐cell responses and sensitising tumours to chemotherapy.^112^

## 5 FUTURE PERSPECTIVE

Chemotherapy has a profound impact on the TME, particularly on B cells. Nevertheless, the effects of chemotherapy on B cells remain understudied. Elucidating the interplay between chemotherapy and B cells, as well as targeting B cells to enhance chemotherapy efficacy, may provide valuable clinical insights. However, numerous questions persist concerning the relationship between chemotherapy and B cells.

### 5.1 Further research on B‐cell functions in the TME

The interactions of B cells within the TME under the influence of chemotherapy warrant further exploration. Multiple studies have demonstrated that NK cells may play a crucial role in inducing antibody secretion by B cells.^113^, ^114^, ^115^ IgG antibodies can be involved in antitumour activity both locally and systemically by activating NK cells through ADCC and macrophages through antibody‐dependent cellular phagocytosis (ADCP).^116^ Deposition of IgG‐containing immune complexes can promote FcγR‐dependent activation of myeloid cells, which may be associated with poor prognosis in tumours.^116^ Apart from T cells, the effects of chemotherapy on other cells within the TME have been insufficiently investigated. The mechanism by which chemotherapy influences the interactions between B cells and nonmalignant cells within the TME, as well as whether the effect of chemotherapy on B cells in the TME is pro‐tumourigenic or anti‐tumourigenic, are topics that warrant further investigation.

### 5.2 B‐cell‐related chemoresistance mechanisms

B cells may contribute to chemoresistance; however, the underlying mechanisms remain elusive. Previous research has primarily focused on tumour cells, often neglecting the critical role of the TME. In a murine model, oxaliplatin treatment led to a significant increase in the number of IgA‐producing PCs expressing PD‐L1, IL‐10 and Fas‐L.^117^ IgA synthesis in PCs is associated with TGF‐β signalling.^118^ Alpha‐smooth muscle actin‐positive (α‐SMA+) myofibroblasts, located in close proximity to IgA+ cells, may serve as a potential source of TGF‐β.^119^ IgA+ PCs induce exhaustion of CD8+ cells and inhibit the activation of antitumour cytotoxic T lymphocytes (CTLs) through the expression of PD‐L1 and IL‐10, leading to oxaliplatin resistance, which can be reversed by B‐cell depletion.^105^, ^120^

XBP1+ B cells are associated with chemoresistance, potentially through the upregulation of IL‐10, endoplasmic reticulum stress responses, and endoplasmic reticulum‐associated degradation (ERAD).^117^ Although the precise mechanism by which IL‐10 promotes tumour cell invasion remains unclear, multiple studies have demonstrated that IL‐10 plays a crucial role in the establishment of an immunosuppressive microenvironment. IL‐10‐producing B cells promote the differentiation of immunosuppressive T cells via TGF‐β1 signalling.^121^ Moreover, matrix metalloproteinases (MMPs) can facilitate the degradation of the ECM, thereby encouraging tumour cell invasion.^122^ IL‐10 has been shown to effectively stimulate macrophages to secrete MMP‐2 and MMP‐9, further enhancing the invasive potential of gastric and colorectal cancer cells.^123^ A cellular state of endoplasmic reticulum (ER) stress can be induced by the microenvironmental characteristics of hypoxia, hypermetabolism and oxidative stress in tumour tissues.^124^, ^125^ A crucial molecule involved in the degradation of misfolded proteins caused by ER stress, DERL3, is primarily enriched in XBP1+ B cells, and the DERL3‐induced ERAD process functions as an oncogenic molecule in the immunosuppressive TME.^126^ However, the precise mechanism underlying DERL3 enrichment in B cells remains unclear.

Investigating B‐cell‐mediated chemoresistance mechanisms may provide insights into strategies for enhancing chemotherapeutic efficacy. However, it remains unclear whether chemotherapeutic agents exert differential effects on B cells through distinct mechanisms associated with B‐cell‐mediated resistance.

### 5.3 TLS function

TLSs are ectopic lymphoid aggregates that form in nonlymphoid tissues. In human malignancies, B lymphocytes are typically localised within the GCs of TLSs. Depending on their maturation stage, B cells express different surface markers such as CD19, CD20 and CD21. Chemotherapy‐induced TLS formation may contribute to anticancer efficacy; however, the precise mechanisms by which TLSs and their resident B cells mediate tumour regression remain to be fully elucidated. In bladder cancer, chemotherapy treatment has been shown to induce the recruitment of B cells and Tfh cells, leading to TLS formation and an inflammatory TME.^127^ Low‐dose cyclophosphamide in combination with CSF1R inhibition has been demonstrated to induce the persistent presence of TLSs containing CD4+CD44+ memory T cells and antigen‐presenting CD86+ B cells at the tumour site, which may be crucial for achieving long‐term disease control.^111^ Neoadjuvant chemoimmunotherapy has the potential to promote TLS maturation, which may be associated with improved disease‐free survival (DFS) in patients with resectable NSCLC.^128^ TLSs exhibit a high degree of immune cell infiltration, including B cells, T cells, macrophages, myeloid DCs and NK cells, which correlates with enhanced sensitivity to chemotherapeutic agents such as gemcitabine, cisplatin, vinblastine and epirubicin.^129^

The roles and therapeutic potential of B cells in TLSs remain to be fully elucidated. TLSs orchestrate immune responses through the formation of organised immune cell aggregates. B cells and their associated pathways facilitate local immune responses within TLSs.^7^ The presence of TLSs and TLS‐associated B cells serves as a prognostic indicator in cancers treated with chemotherapy.^130^, ^131^, ^132^, ^133^, ^134^ The maturation of TLSs, as evidenced by the formation of GCs, is associated with further improvements in clinical outcomes.^135^, ^136^ Nevertheless, the precise roles of B cells within TLSs remain incompletely understood. The majority of mouse models employ cultured cell lines, which infrequently develop spontaneous TLSs, necessitating investigations into the potential impact of this limitation on TLS research.^137^ While promoting TLS formation and activity through chemotherapy has been shown to enhance therapeutic efficacy in preclinical models,^138^ clinical trials are warranted to validate these findings.^137^

### 5.4 B‐cell heterogeneity and spatial heterogeneity

The heterogeneity of B cells warrants further investigation. During tumour progression, B cells proliferate and acquire molecular and genetic alterations that contribute to heterogeneity, which in turn affects tumour growth, invasion, drug sensitivity and prognosis. Specific B‐cell subpopulations have been shown to predict responses to chemotherapy and immunotherapy and display various interactions within the TME.^139^, ^140^ Promoting anti‐tumour B‐cell subtypes may enhance anti‐tumour immunity.^141^ Certain subpopulations are resistant to anti‐CD20 depletion, suggesting that a combination of CD20 antibody and anthracycline therapy may be warranted.^142^ Characterising B‐cell heterogeneity may facilitate the development of personalised treatment strategies.

Spatial heterogeneity of B cells may also serve as a predictor of survival; however, standardised metrics for its assessment are currently lacking. In a study on lung cancer, quadratic entropy was employed to quantify spatial heterogeneity, revealing that it limited the associations between CD20+ B cells and survival.^143^ Metrics that accurately capture spatial heterogeneity could guide treatment regimens and help avoid overtreatment.^143^, ^144^ Regions of the TME with high heterogeneity may contain subclones that are resistant to treatment.^143^ The development of standardised metrics for spatial heterogeneity is crucial.

### 5.5 The selectivity of chemotherapy on B cells

Different chemotherapy drugs act selectively on B cells, leading to variations in B‐cell function recovery. Researchers often overlook the distinctions between chemotherapy drugs and generally refer to them as ‘chemotherapy’. However, our findings suggest that different classes of chemotherapy drugs can lead to disparities in B‐cell recovery. Anthracycline‐based regimens caused a more profound reduction in B cells compared to anthracycline‐paclitaxel sequences, although the latter delayed B‐cell recovery.^26^ Methotrexate and cyclophosphamide had a notable impact on MZ‐B cells. Despite minimal changes in splenic cell counts, methotrexate inhibited B‐cell function, diminishing responses to TI‐2 antigens without impacting T‐cell‐dependent responses.^88^ Cisplatin had a minimal effect on B‐cell counts and immune responses.^88^ Combination chemotherapy (cyclophosphamide, vincristine, prednisone, doxorubicin and L‐asparaginase) depleted circulating B cells but not those in lymph nodes, thus preserving antibody production.^145^ Methotrexate, Cisplatin, paclitaxel and 5‐fluorouracil inhibited B‐cell proliferation to varying extents, with CP nearly completely blocking it.^94^ These findings suggest that variations in adaptive immunity, mediated by the influence on B cells, may result in different chemotherapy effects. Future studies that differentiate between various types of chemotherapy drugs may yield more accurate results.

### 5.6 Further study of B‐cell targets

At present, B‐cell‐related targets show great potential and are garnering increasing interest in anti‐tumour therapy; however, two significant challenges persist. First, investigations of B‐cell‐related targets in solid tumours remain restricted to animal models and in vitro cell lines. Second, although B‐cell‐related targeted medications are currently undergoing clinical trials, it has yet to be determined whether targeted therapy combined with chemotherapy demonstrates superior clinical anti‐tumour efficacy compared to targeted therapy or chemotherapy alone. Several targeted therapies for BTLA, such as TAB004, JS004 and HFB200603, are currently in clinical trials.^107^, ^146^, ^147^, ^148^ While the combination of chemotherapy and anti‐BTLA antibodies has demonstrated enhanced anti‐tumour efficacy in mouse models, it remains uncertain whether targeted BTLA therapy in conjunction with chemotherapy enhances anti‐tumour efficacy in humans. Anti‐CD55 (decay‐accelerating factor, DAF) therapy has been shown to synergistically enhance the tumouricidal and antimetastatic effects of 5‐Fluorouracil in colorectal cancer cells, suggesting that combination therapy may be a superior treatment approach for colorectal cancer; however, further animal studies and clinical trials are necessary to confirm this.^149^ Targeted agents against BTK, including FDA‐approved Ibrutinib, Acalabrutinib, Zanubrutinib, Tirabrutinib, and Orelabrutinib, as well as Pirtobrutinib, which is currently undergoing clinical trials, have demonstrated potent therapeutic efficacy in various B‐cell malignancies; however, their therapeutic potential in solid tumours warrants further investigation.^150^ Currently, most studies on BTK‐targeted therapy in solid tumours have been limited to animal models and in vitro cell lines. Interestingly, targeting BTK has demonstrated anti‐tumour effects in pancreatic ductal adenocarcinoma (PDAC) homozygous mice.^112^ Moreover, clinical trials have been conducted to evaluate the efficacy of BTK inhibitors in solid tumours, and the results are worth looking forward to. Furthermore, WNT16B and Rab27a have emerged as potential therapeutic targets; however, their roles in anti‐tumour resistance and development remain to be elucidated.

In summary, investigating the chemotherapy–B‐cell interface emphasises the anti‐tumour potential of B cells. Modulating B‐cell functions through targeted therapies could enhance chemotherapy efficacy. However, critical knowledge gaps persist concerning B‐cell interactions within the TME, B‐cell chemoresistance mechanisms, tertiary lymphoid structure biology, heterogeneity, spatial distributions, chemotherapy drug selection and B‐cell‐related targets, which warrant further exploration in future studies.

## AUTHOR CONTRIBUTIONS

*Zizhuo Li*: Conceptualised the article, performed the literature search and wrote the manuscript. *Anqi Lin*: Conceptualised the article and reviewed the manuscript. *Zhifei Gao*: Conceptualised the article and reviewed the manuscript. *Aimin Jiang*: Complemented the manuscript. *Minying Xiong*: Complemented the manuscript. *Jiapeng Song*: Complemented the manuscript. *Zaoqu Liu*: Complemented the manuscript. *Quan Cheng*: Conceived the study content and provided constructive guidance. *Jian Zhang*: Conceived the study content and provided constructive guidance. *Peng Luo*: Conceived the study content and provided constructive guidance. All of the authors have read and approved the final manuscript.

## CONFLICT OF INTEREST STATEMENT

The authors declare no conflicts of interest.

## ETHICS STATEMENT

Not applicable.
